# Extreme-aged patients (≥ 85 years) experience similar outcomes as younger geriatric patients following chronic subdural hematoma evacuation: a matched cohort study

**DOI:** 10.1007/s11357-024-01081-8

**Published:** 2024-01-30

**Authors:** Peyton L. Nisson, John J. Francis, Michelot Michel, Keshav Goel, Chirag G. Patil

**Affiliations:** 1 Department of Neurosurgery, Cedars-Sinai Medical Center, Los Angeles, CA USA; 2 School of Medicine, Case Western Reserve University, Cleveland, OH USA; 3 College of Medicine, University of Florida, Gainesville, FL USA; 4 David Geffen School of Medicine, University of California Los Angeles, Los Angeles, CA USA

**Keywords:** Subdural hematoma, Geriatric, Subdural hematoma evacuation, Age, Nonagenarians, Risk factor, Octogenarian

## Abstract

Subdural hematoma (SDH) evacuation represents one of the most frequently performed neurosurgical procedures. Several reports cite a rise in both the age and number of patient’s requiring treatment, due in part to an aging population and expanded anticoagulation use. However, limited data and conflicting conclusions exist on extreme-aged geriatric patients (≥ 85 years of age) after undergoing surgery. Patients undergoing SDH evacuation at a tertiary academic medical center between November 2013-December 2021 were retrospectively identified. The study group consisted of patients ≥ 85 years (Group 1) diagnosed with a chronic SDH surgically evacuated. A control group was created matching patients by 70–84 years of age, gender, and anticoagulation use (Group 2). Multiple metrics were evaluated between the two including length-of hospital-stay, tracheostomy/PEG placement, reoperation rate, complications, discharge location, neurological outcome at the time of discharge, and survival. A total of 130 patients were included; 65 in Group 1 and 65 in Group 2. Patient demographics, medical comorbidities, SDH characteristics, international normalized ratio, partial thromboplastin time, and use of blood thinning agents were similar between the two groups. Kaplan Meier survival analysis at one-year was 80% for Group 1 and 76% for Group 2. No significant difference was identified using the log-rank test for equality of survivor functions (*p* = 0.26). All measured outcomes including GCS at time of discharge, length of stay, rate of reoperations, and neurological outcome were statistically similar between the two groups. Backwards stepwise conditional logistic regression revealed no significant association between poor outcomes at the time of discharge and age. Alternatively, anticoagulation use was found to be associated with poor outcomes (OR 3.55, 95% CI 1.08–11.60; *p* = 0.036). Several outcome metrics and statistical analyses were used to compare patients ≥ 85 years of age to younger geriatric patients (70–84 years) in a matched cohort study. Adjusting for age group, gender, and anticoagulation use, no significant difference was found between the two groups including neurological outcome at discharge, reoperation rate, and survival.

## Introduction

Subdural hematomas (SDH) represent a growing public health concern in the United States and world at large. Elderly patients are both at a higher risk for experiencing fall related SDHs and mortality compared to younger adults [1]. According to U.S. Census bureau, the number of people > 85 years of age increased from 3 million to 4.3 million (43% increase) from 1999 to 2008 [2]. Current projections indicate individuals ≥ 65 years will constitute over 20% (73 million) of the U.S. population by 2030 [3]. The United Nations estimate 1.5 billion individuals of the world population will be over 65 by 2050, with those over 80 representing the fastest growing segment of the population in developed countries [4].

The expansion of blood thinning medication use over the past 3 decades has only exacerbated the frequency and severity of SDHs seen in the elderly [5, 6]. Between 1988 and 1999 warfarin administration quadrupled, which was paralleled by a quintuple incidence of anticoagulation-associated intracerebral hemorrhage [7]. Implementation of the CHA2DS2-VASc score in 2001 for stroke prevention categorized older age (≥ 75 years) alone as a risk factor and indication for anticoagulation use [8]. Prescriptions have since increased with a 16-fold increase in Medicare spending in oral AC use from 2011 to 2019 [9–11]. Confounding this issue are the rising rates of neurodegenerative diseases, with an estimated quadruple prevalence of Alzheimer disease by 2050. These patients are at a 2 to threefold increased risk for falls, who may also be on blood-thinning medications [12, 13].

These findings coincide with a rising incidence of SDH evacuations across medical centers, with those of advanced age most effected [14, 15]. As such, there exists a need to address this emerging public health issue and improve patient outcomes [16]. Currently, conflicting findings exist with some studies citing very high rates of mortality and poor neurological outcomes [17–20]. There is concern for the role of surgical intervention in extreme-aged geriatric patients who often possess a disproportionately high number of co-morbidities, frailty, and shorter life expectancy than those younger [21]. Similarly, families often express concern for their relative’s ability to tolerate a procedure as invasive as brain surgery at their age. Patient’s ≥ 85 years represent one of the most extreme age groups studied on this topic and most effected by the physiologic changes of aging. To date, only a limited number of studies have investigated outcomes in this group or older, most of which have been descriptive analyses [17–20, 22–24]. Only one has performed a Kaplan–Meier survival analysis accounting for patients lost to follow up [23]. The largest series ever analyzed consisted of 75 patients in Scandinavia 90 years or older [24]. In this current study, we compared the multiple surgical outcomes of patients with subdural hematomas 85 years or older to those of younger geriatric patients (70–84 years) using a matched cohort study design (adjusting for age, gender, and anticoagulation use) to further delineate the effect advanced age has on surgical outcomes.

## Methods

Consecutive patients who underwent SDH evacuation between November 2013 and December 2021 at a single, tertiary academic medical center were retrospectively identified using International Classification of Diseases-9 and -10 billing codes. Patients with an age 70 years or older at the time surgery were identified. Initially this comprised 352 records, 87 of which were duplicates, or patients had an epidural hematoma, parenchymal hemorrhage, or no surgery was performed. Five patients who underwent craniectomies for cerebral edema were excluded from the analysis. Subdural hematomas were defined as chronic based on the presence of a primary hypodense, isodense, or mixed-density subdural collection. Patients with a hyperdense fluid collection were categorized as acute SDHs and subsequently excluded from the study (*n* = 59). Patients ≥ 85 years of age were categorized as Group 1 (*n* = 65). These patients were then matched 1:1 to a younger cohort of patients between 70–84 years, along with gender and anticoagulation use (Group 2).

Data was retrieved from the electronic medical record including basic demographics, pertinent past medical history, international normalized ratio (INR), partial thromboplastin time (PTT), and platelets at time of hospital admission arrival, current use of blood thinning agents, SDH features, and surgery type. Surgery types were categorized as craniotomy or burr hole craniostomy. Placement of a subdural or subgalea drain intra-operatively was performed at the discretion of the attending neurosurgeon, commonly weighing factors in this decision such as infection risk, dryness of the subdural space at the time of closure, perceived risk for recurrence, and patient preference/cooperativeness for an indwelling drain. Drains were typically removed on post-operative day 2 or 3 and or when output was less than 50 cc over a 24-h time period. No SDH evacuation port systems were offered or used in this study.

Outcomes were compared between the two groups using the rate of complications, tracheostomy/percutaneous endoscopic gastrostomy (trach/PEG), length of stay (LOS), reoperation rate, discharge location, inpatient mortality, 30-day mortality, survival estimates, and neurological exam at discharge. Neurological exams were reported using the Glasgow Coma Scale. The Glasgow Outcome Scale (GOS) was also used to dichotomize outcomes as either favorable (grades 4–5) or poor (grades 1–3). Data available on patient follow-up was retrieved up to 5 years following the time of SDH evacuation.

### Statistical Analysis

Patient demographics and outcomes were compared between the groups using *X*^2^ for categorical variables; if the expected frequency for an observation was less than 5 the Fisher exact test was used. Continuous data was compared using the student’s t-test. Kaplan–Meier Survival estimates were performed with death as censored data and log-rank test of equality between the two age groups. Backwards stepwise conditional logistic regression analysis was performed using “poor outcome” at time of discharge at the outcome variable and inclusion criteria set a p-value of less than 0.20. International normalized ratio, PTT, and atrial fibrillation were not included due to concern for interaction with the variable anticoagulation, which was instead included in the construction of the model. Only a p-value equal or less than 0.05 was considered significant. Any missing observations were left blank during analysis. Statistical analysis was performed using STATA 14 (StataCorp LP, College Station, Texas).

## Results

A total of 130 patients were included in the study; 65 patients were ≥ 85 years of age (Group 1) and were matched with 65 patients 70–84 years of age (Group 2). Age ranged from 70 to 100 years with a mean of 83 years. Mean subdural size was 21.8 mm (Range 7-40mm). Ground-level fall was the most common mechanism for traumatic injury, accounting for 50% (*n* = 65) of patient presentations. Glasgow coma scale scores upon arrival were the following: 0% between 3–8, 3% (*n* = 4) between 9–12, 97% (*n* = 126) between 13–15. The majority of patients had a subdural drain placed following hematoma evacuation (87%, *n* = 113). There were 4 patients who also had middle meningeal artery embolization (3%) in the post-operative period. No differences were present in co-morbidities, GCS score on arrival, SDH size, midline shift, or INR, PTT, or platelets at the time of arrival. Further detail summarizing these two groups are included in Table 1.

**Table 1:** Patient demographic, medical history, and presentation summary. Abbreviations: ASA; aspirin, GCS; Glasgow Coma Scale

Characteristics	≥ 85	70–84	P-value	Total
Number of patients	65	65	-	130
Median Age (years)	89	77	-	85
Age Range (years)	85–100	70–84	-	70–100
Males	47 (72)	47 (72)	1	94 (72)
Medical Comorbidities				
Hypertension	34 (52)	31 (48)	0.73	66 (50)
Diabetes Mellitus	9 (14)	16 (24)	0.6	65 (50)
Coronary Artery Disease	16 (25)	12 (19)	0.39	28 (22)
Cerebrovascular Accident	3 (5)	7 (11)	0.19	10 (8)
Congestive Heart Failure	6 (9)	7 (11)	0.77	13 (10)
Atrial Fibrillation	13 (20)	11 (17)	0.65	24 (19)
Mean SDH size mm (SD)	22.2 (7)	21.4 (7)	0.51	21.8 (7)
Bilateral SDH Present	17 (26)	18 (28)	0.46	35 (27)
Mean Midline Shift mm (SD)	5.6 (4)	6.4 (5)	0.38	6 (5)
Surgery Type				
Craniotomy	52 (80)	56 (86)	0.35	108 (83)
Burr Hole	13 (20)	9 (14)		22 (17)
INR at Arrival (SD)	1.2 (0.5)	1.3 (0.7)	0.35	1.3 (0.6)
PTT at Arrival (SD)	31.9 (9)	32.4 (9)	0.74	32.1 (9)
Platelets at Arrival (SD)	205 (75)	231 (78)	0.06	218 (78)
Antiplatelet Medication Use	29 (45)	27 (41)	0.72	56 (43)
ASA (Y)	27 (42)	25 (39)	0.67	52 (40)
Clopidogrel/Ticagrelor (Y)	10 (15)	8 (12)	0.61	18 (14)
Anticoagulation Use (Y)	7 (23)	7 (23)	1	21 (23)
GCS at time of Arrival				
3 to 8	0	0	0.62	2 (2)
9 to 12	3 (2)	1 (2)		4 (3)
13 to 15	62 (95)	64 (98)		126 (97)

### Outcomes

The rate of re-operation following subdural hematoma evacuation was 15% (*n* = 19) and infections occurred in 4% (*n* = 5) of cases. Inpatient mortality was 5% (*n* = 7) and 6% (*n* = 8) at 30 days after discharge. The mean follow up was 1.7 years (Range 0 to 5 years). Statistical analysis revealed no difference in GCS at the time of discharge, perioperative complications, trach/PEG placement, LOS, discharge location, rate of poor outcomes or mortality between the age groups (Table 2**)**.

**Table 2:** Patient outcomes were compared between age groups. Poor Outcomes were defined by a Glasgow Outcome Scale 1 to 3 at the time of discharge. Abbreviations: GCS; Glasgow Coma Scale, GOS: Glasgow Outcome Scale, DVT; deep vein thrombosis, PE; pulmonary embolism, PEG; percutaneous endoscopic gastrostomy tube, SD; standard deviation

Outcomes	≥ 85	70–84	P-value	Total
GCS at Discharge				
3 to 6	4 (6)	2 (3)	0.08	6 (5)
7 to 12	2 (3)	0		2 (2)
13 to 15	56 (86)	63 (97)		119 (92)
Complications				
Urinary Tract Infection	2 (3)	2 (3)	1	4 (3)
Pneumonia	2 (3)	0	0.5	2 (2)
DVT/PE	0	0	1	0
Infection	3 (5)	2 (3)	1	5 (4)
Seizure	5 (8)	2 (3)	0.44	7 (5)
Stroke	1 (2)	1 (2)	1	2 (2)
Trach/PEG Placed (Y)	5 (8)	4 (6)	1	9 (7)
Average Length of Stay Days (SD)	10.5 (11)	9.3 (6)	0.44	9.9 (9)
Reoperation	10 (15)	9 (14)	0.8	19 (15)
Discharge Location				
Nursing facility	7 (11)	10 (15)	0.41	17 (13)
Rehab	33 (52)	26 (40)		59 (46)
Home	20 (31)	27 (42)		47 (36)
Hospice	4 (6)	2 (3)		6 (5)
Poor Outcome at Discharge (GOS 1–3)	10 (15)	4 (6)	0.09	14 (11)
Mortality				
Inpatient Mortality	5 (8)	2 (3)	0.44	7 (5)
30-Day Mortality	6 (9)	2 (3)	0.27	8 (6)
1-Year Survival	76%	80%	-	78%
Mean Follow up (years)	1.7 (1.9)	1.7 (1.8)	1	1.7 (1.8)

### Survival and Logistic Regression Analysis

**Fig. 1:**
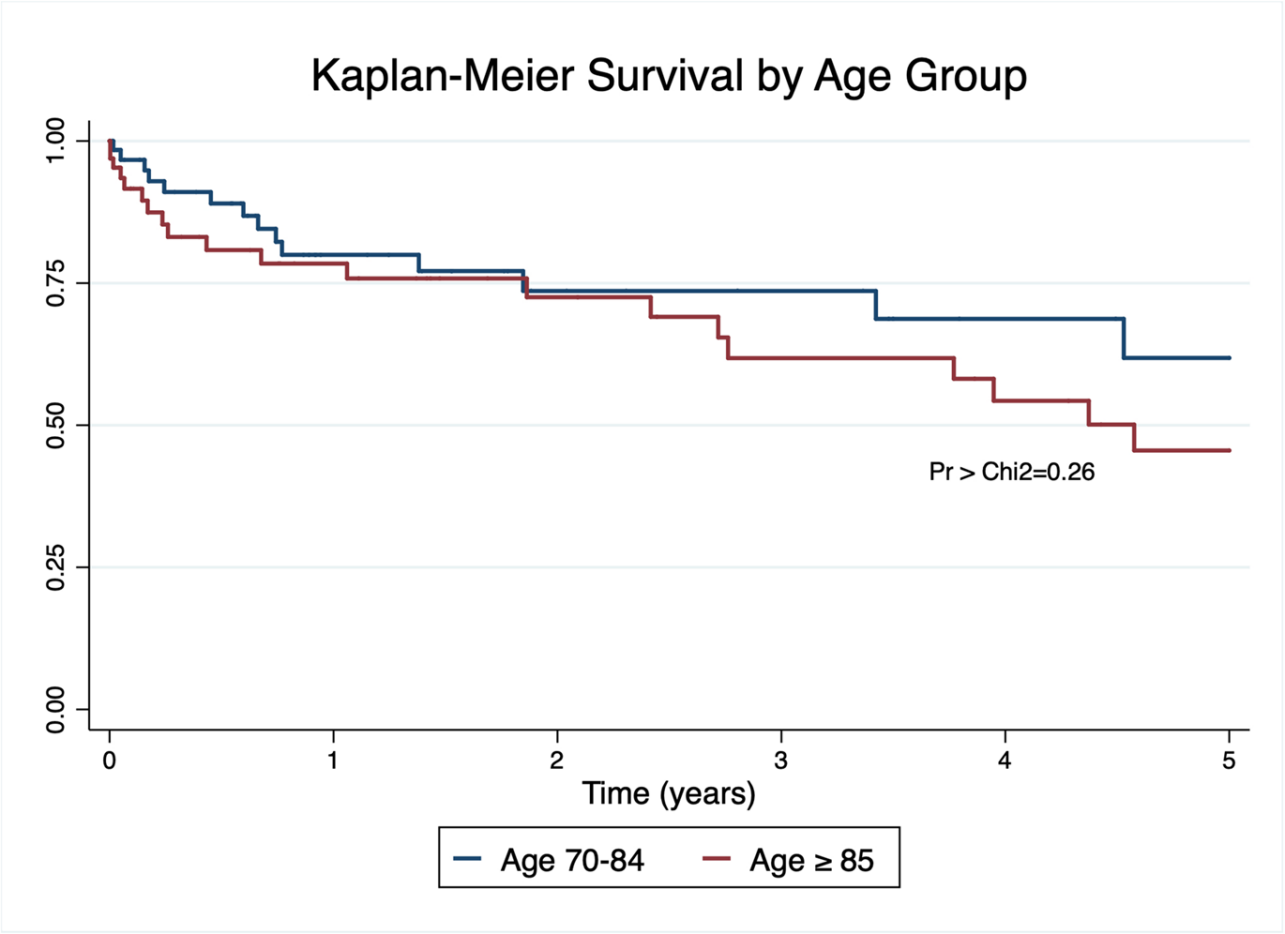
Kaplan–Meier 5-year survival estimates compared between age groups. Log-rank test of equalities showed no difference (*p* = 0.26) between patients ≥ 85 years of age (Group 1) to those 70–84 years (Group 2)

Kaplan–Meier survival analysis revealed 1-year survival was 76% for Group 1 and 80% for Group 2. The log-rank test for equality of survivor functions revealed no significant difference in survival (*p* = 0.26), as shown in Fig. 1. Backwards stepwise conditional logistic regression analysis was then performed with poor neurological outcome set as the dependent variable. Patient gender, age (as a continuous variable), HTN, DM, SDH size, midline shift size, presence of bilateral SDHs, anticoagulation use, antiplatelet use, and platelets at the time of arrival were included. Among these, anticoagulation (OR 3.6, CI 1.08–11.60, *p* = 0.036) was significantly associated with poor outcomes. Midline shift (OR 1.1, CI 0.95–1.26, *p* = 0.186) and platelets at the time of arrival (OR 0.99, 95% CI 0.99 – 1.00, *p* = 0.18) met inclusion criteria for the model but were non-significant.

## Discussion

Extreme-aged geriatric patients (≥ 85 years) demonstrated no significant difference in neurological outcome, complications, discharge location or survival compared to younger geriatric patients (70–84 years) using a matched cohort study design. Anticoagulation use (OR 3.6, CI 1.08–11.60, *p* = 0.036) was associated with poor neurological outcomes in the multivariable regression analysis which has been previously associated with a higher morbidity and mortality [25]. With the exception of the study by Dumont et al. (2013), this analysis represents one of the only statistical comparisons conducted on patients ≥ 85 years to another age group in the United States and the third largest cohort of patients analyzed on the elderly population in this age group or older [14, 17–19, 22–24, 26–30]. Although limited research exists on this unique population, it remains an active area of interest in light of projected population trends [31, 32]. Review of the literature (Table 3) revealed 7 other articles that have reported specifically on patients ≥ 85 years of age [17–20, 22–24]. Among these, mortality rates varied widely, from 0 to 31% at the time of discharge [17, 19]. Our findings corroborate the results of Bartek et al. (2017) and Dorban et al. (2022) who found no significant difference in outcomes for patients in their cohorts ≥ 90 years of age with chronic SDHs evacuated compared to younger patients [18, 24].

**Table 3:** Literature Review of Studies on surgically evacuated subdural hematomas in the elderly (≥ 65 years) published since the year 2000. SC; single center. MC; multicenter. GOS; Glasgow outcome scale. mRS; modified Rankin Scale. NA; not available. MKS; Markwalder grading scale

Author/Year	Location	Study Type	Data Date Range	Age Group (years)	Chronicity	# of Patients	Functional Outcome	Mortality
Asghar et al.[26] 2002	UK	MC Retrospective Analysis	1996–1999	> 65	Chronic	24	NA	17% inpatient
Liliang et al.[48] 2002	Taiwan	SC Retrospective Analysis	1995–2000	> 75	Chronic	51	46% “improved”	1.9% inpatient
		SC Retrospective Analysis	1995–2000	< 40	Chronic	24	23% “improved”	0% inpatient
Miranda et al.[27], 2011	USA	Prospective Database	2000–2008	≥ 65	Chronic	137	NA	26.3% at 6 months (includes non-operative patients)
Stippler et al.[19], 2013	USA	SC Retrospective Analysis	2005–2011	≥ 90	Chronic	16	NA	31% inpatient
Dumont et al.[23], 2013	USA	SC Retrospective Analysis	1996–2010	55–64	Chronic	32	NA	9% inpatient
		SC Retrospective Analysis	1996–2010	65–74	Chronic	51	NA	6% inpatient
		SC Retrospective Analysis	1996–2010	75–84	Chronic	62	NA	10% inpatient
		SC Retrospective Analysis	1996–2010	≥ 85	Chronic	21	NA	12% inpatient
Tabuchi and Kadowaki.[17], 2014	Japan	SC Retrospective Analysis	2007–2013	> 90	Chronic	12	100% mRS < 2.5 at discharge/1 month	0% inpatient
Whitehouse et al.[28], 2016	UK	SC Retrospective Analysis	2007–2010	≥ 75	Acute + Chronic	263	(GOS 1–3) 25% at discharge	7.7% inpatient
Lee et al.[22], 2016	Singapore	MC Retrospective Analysis	2001–2013	≥ 90	Chronic	70	NA	7.1% at 30 days
							NA	18.6% at 6 months
Bartek et al.[24] 2017	Scandinavia	MC Retrospective Analysis	2005–2010	≥ 90	Chronic	75	(Landriel-Ibanez Grade ≥ 3) 1.4% at 30 days	4% at 30 days
								13.3% at 90 days
				< 90	Chronic	1179	(Landriel-Ibanez Grade ≥ 3) 3.1% at 30 days	3.3% at 30 days
							NA	5.7% at 9 days
Christopher et al.[20] 2018	UK and Ireland	MC Prospective Analysis	2013–2014	≥ 90	Chronic	68	(mRS 4–6) 41.2% at discharge	8.8% inpatient
				< 90	Chronic	696	(mRS 4–6) 20.5% at discharge	1.1% inpatient
Dobran et al.[18], 2022	Italy	SC Retrospective Analysis	2012–2016	≥ 90	Chronic	25	MKS (0–1) 100% at 1 month	4% at 30 days
		SC Retrospective Analysis					MKS (0–1) 100% at 6 months	4% at 6 months
		SC Retrospective Analysis	2012–2016	75–85	Chronic	25	MKS (0–1) 96% at 1 month	0%
		SC Retrospective Analysis					MKS (0–1) 96% at 6 months	0%
Sunblom et al.[30], 2022	Sweden	SC Retrospective Analysis	2010–2014	≥ 70	Chronic	511	MKS (0–1) 86.3% at discharge	3.1% at 30 days
Current Series	USA	SC Retrospective Analysis	2013–2021	≥ 85	Chronic	65	GOS (1–3) 15% at discharge	8% inpatient
				70–84	Chronic	65	GOS (1–3) 6% at discharge	3% inpatient

### Subdural Hematoma Evacuation Management

When considering an alternative to surgery, patients may potentially be subjected to an indefinite period of debilitating headaches, seizures, hemiparesis, aphasia, gait imbalance, or possible death, depending on the presenting symptoms. Surgical evacuation remains the gold standard for curation while medical management is unfortunately limited primarily to symptom alleviation. Patients treated with surgery on average survive over twice as long as those without (2.1 vs. 1.0 years for patients ≥ 85 years) [23]. This must be weighed against the risk of exposing patients to unnecessary procedures that might only prolong or worsen suffering and not improve outcomes. Though age is often used as a surrogate for frailty, these two are not interchangeable. Frailty is unique to each patient and likely represents a more accurate metric for evaluating the ability to tolerate surgery as opposed to age alone [33]. In the trauma patient population, a recent systematic review and meta-analysis by Alqarni et al. (2023) found frailty to be a superior predictor for adverse outcomes than age in the geriatric population [34]. Previously proposed scoring systems for frailty have shown promise in the SDH population [35, 36]. Some limitations though of frailty assessment scores have been an inherent degree of subjectiveness in their design with limited interrater reliability, particularly between specialties [37].

Surgical management of SDHs in the geriatric population has not been without controversy [38]. Studies citing a high rate of mortality and poor outcomes have raised concern for the utility of intervention. In a recent systematic review, 81% of patients with acute SDHs evacuated experienced a poor outcome (GOS 1–3) and 49% expired at the time of most recent follow-up [21]. The United Kingdom-based study by Whitehouse et al. (2016) reported 15 times greater odds for inpatient mortality in those with chronic SDHs ≥ 75 years of age [28]. Dumont et al. (2013) found the shortest mean survival for patients ≥ 85 years after chronic SDH evacuation [23]. Conversely, Ramachandran and Hegde (2007) reported favorable outcomes (GOS 4–5) in 75% over 60 years of age with chronic SDHs evacuated [39]. Tabuchi and Kadowaki (2014) reported no deaths in their series of 12 patients with 66.7% returning directly home from the hospital [17]. More recently, Dobran et al. (2022) found 100% of patients in their ≥ 90 years group experienced favorable outcomes (Markwalder grading scale [MKS score] 0–1) [18]. Sundblom et al. (2022) also reported 86.3% had favorable outcomes with MKS score between 0–1 [30].

An important factor complicating the replicability of outcomes is how heterogenous SDHs are as a group. Frequently occurring in the setting of traumatic brain injury, they can be accompanied by other sequalae including cerebral edema, intraparenchymal hemorrhages, diffuse axonal injury, and subarachnoid hemorrhages. Alternatively, they can occur insidiously with no known history of trauma or inciting event. Patients may or may not be taking an anticoagulant. The location and surrounding populous of a medical center can greatly influence the referral pattern-for instance a hospital in a large metropolitan area located near a major highway versus one in a smaller retirement community. The standards and medical practices across countries can also differ greatly [40].

Altogether, these features make this relatively common condition a challenge to study and may account for some of the large variation in outcomes with inpatient mortality rates (0%-31%) [14, 21, 28, 38, 41]. In this investigation, any patients who underwent decompressive hemicraniectomies and or intraparenchymal hematoma evacuations were excluded in an effort to avoid other cerebral pathologies that may influence outcomes. In part, this could account for a difference in outcomes seen in this series to those reported from other studies [19, 23]. Future investigation for the development of a more specific and standardized classification scheme for SDHs would be valuable in addressing this.

### Extreme-Aged Geriatric Patients and Effects of Ageing

It is well understood that aging is marked by a gradual decrease in cellular robustness. This complex process is attributed, in part, to telomere shortening, DNA damage from free radicals and radiation, accumulation of glycosylation end-products and protein aggregates, and cessation of cellular proliferation [42, 43]. On a macro level these cumulative changes can be recognized as skeletal muscle mass wasting, atherosclerotic disease, cerebral atrophy, and weakening of the immune system, to name a few [44, 45]. Independent of any particular surgery type or indication, elderly patients are at a known higher risk for cardiac and non-cardiac complications [46]. As such, patients in the ≥ 85 age group would be expected to likely experience an increased risk for mortality and poor outcomes. Yet, our results add to the growing literature that has reported a similar outcome profile as younger aged geriatric patients [17, 18, 24].

These seemingly paradoxical findings suggest potentially competing phenomena offsetting the deleterious physiologic changes of aging. We suspect 1) there may be stricter screening process for surgical candidacy in patients, with more families pursuing conservative management, comfort care, or physicians not recommending surgery for patients who they may have otherwise been more aggressive with had they been a decade younger. 2) Health care providers (i.e., anesthesia, nursing, surgeon, etc.) may unknowingly practice greater vigilance for complication avoidance in this seemingly higher risk age group. 3) Patients in the 85 or above age group represent even greater outliers from the general population compared to their younger geriatric counterparts and likely exhibit a combination of health-based practices and or genetic predisposition for greater than normal longevity; surpassing the life expectancy age of 76.1 years by nearly a decade in the United States per CDC estimates (2022) [47]. In turn, this may translate into improved tolerance for injury associated with SDHs, risks of surgery, and overall survival time.

### Limitations

Limitations of this investigation include its retrospective study design. The patient referral pattern and surrounding socioeconomic demographics of this tertiary academic institution may influence the generalizability of the findings. It is possible the ≥ 85 years age group was not large enough in sample size to capture a significant difference between the two groups. Patients lost to follow-up may also cause some underestimation for mortality. In the survival analysis we attempted to control for this using death as a censored data function.

## Conclusion

Extreme-aged geriatric patients represent a growing segment of the population who are disproportionately prone to SDHs. Reports of alarming high complication rates and poor outcomes has raised concern for the efficacy of surgical intervention. In this study patients ≥ 85 years with chronic SDHs exhibited no difference in complications, recurrence, mortality, or good functional outcomes at 5-year follow-up. Kaplan–Meier survival analysis and logistic regression analysis both revealed no difference in survival or neurological functional outcome between age groups. When assessing surgical candidacy in the geriatric population, it is important to consider co-morbidities, life expectancy, and overall health status. Though age is a pertinent factor in this assessment, among the geriatric population, no added risk was found in those ≥ 85 compared to younger geriatric patients following surgical evacuation of hematomas.
